# Incompatibilities in Prescriptions

**Published:** 1892-12

**Authors:** E. B. Stuart


### INCOMPATIBILITIES IN PRESCRIPTIONS.

Some time ago a prescription was sent to me from one of the Southern States, by a former pupil. If the object of the author of this prescription was to crowd as many incompatibilities as possible into a short prescription, he has succeeded pretty well.

The reactions occurring when this prescription is compounded are simple, and should be understood by any student who has taken a college course in chemistry, but the prescription is an interesting one from the number of distinct incompatibilities it presents, there being about as many as there are ingredients in the mixture. It is as follows:

| | |
|---|---|
| Tincturæ ferri chloridi ........... ............ | ʒ iss. |
| Sodii hyposulphitis ........................... | ʒ iss. |
| Potassii chloratis .............................. | ʒ iij. |
| Quininæ sulphatis ............................ | gr. xv. |
| Aquæ ............................................. | ℥ ij. |

The original dispenser mixed the several articles in the order in which they are written above, save the water, a portion of which was used to dissolve the sodium thiosulphate. If the several reactions which occur in this case are noted, they will be found to be as follows: On adding the solution of the thiosulphate to the tincture of iron, the brown color of the latter is quickly changed to the pea-green characteristic of ferrous compounds, and, at the same time, a slight milkiness, due to the separation of sulphur, occurs.

The ordinary tests for ferric iron show that all the ferric salt has been reduced to ferrous. At this point, the mixture probably contains ferrous chloride, sodium sulphate and sodium thiosulphate, the reaction being as follows: $2Fe_2Cl_6 + 2Na_2S_2O_3 + 2H_2O = 4FeCl + 2Na_2SO_6 + 4HCl + S_2$.

The hydrochloric acid set free is immediately decomposed by the thiosulphate, with the evolution of $SO_2$.

These two decompositions leave about sixty-six grains of sodium thiosulphate still undecomposed.

On adding the potassium chlorate (180 grains), this remaining quantity of thiosulphate is entirely decomposed, and the ferrous chloride again raised to ferric chloride. One molecule of potassium chlorate will give up enough oxygen to convert six molecules of ferrous chloride into ferric salt, and six molecules of free hydrochloric acid will be required to satisfy the increased valence of the ferric iron. Inasmuch as the acid set free by the reduction of the ferric chloride originally used was destroyed by the thiosulphate, at least one-third of the iron will be precipitated as oxide, as shown by the following equation: $6FeCl_2 + KClO_3 = 2Fe_2Cl_6 + Fe_2O + KCl$, or, more probable, two-thirds of the iron will be precipitated as oxychloride, and one-third remain in solution as chloride. In either case, the amount of potassium chlorate decomposed is the same, amounting to only about one and one-half grains, and leaving an abundant supply for the decomposition of the sixty-six grains of sodium thiosulphate which still remained, and which is promptly oxidized to sulphate, as follows: $6Na_2S_2O_3 + 2KClO_3 = 6Na_2S_2O_4 + 2KCl + 3S_2$. Not quite eleven grains of potassium chlorate is required to decompose the above quantity of crystallized sodium thiosulphate, making a total loss of chlorate of about twelve and one-half grains, and leaving a large excess for the next reaction, which occurs after the quinine is added, when, owing to the insolubility of the chlorate of this alkaloid, it exchanges its acid radical with the potassium chlorate, forming, of course, an equivalent quantity of potassium sulphate. This is especially noticeable if the quinine salt is dissolved before adding it to the mixture.

Our curious and unusual series of reactions is now complete, and the vial contains ferric chloride and oxychloride, precipitated sulphur, sodium sulphate and chloride, potassium sulphate chloride and chlorate, and quinine chlorate. The question as to what the physician who is responsible for this aggregation of incompatibilities intended to administer, remains a mystery; that of the expected effect upon the patient has excited the curiosity of several members of the profession. With these questions the pharmacist is, perhaps, not legitimately concerned, and *the average medical student continues to look upon the time he is obliged to spend in the study of chemistry while at college as simply wasted.*—E. B. STUART, in *The Apothecary.*

